# A Ligation/Recombinase Polymerase Amplification Assay for Rapid Detection of SARS-CoV−2

**DOI:** 10.3389/fcimb.2021.680728

**Published:** 2021-05-28

**Authors:** Pei Wang, Chao Ma, Xue Zhang, Lizhan Chen, Longyu Yi, Xin Liu, Qunwei Lu, Yang Cao, Song Gao

**Affiliations:** ^1^ Key Laboratory of Molecular Biophysics of Ministry of Education, Department of Biomedical Engineering, College of Life Science and Technology, Center for Human Genome Research, Huazhong University of Science and Technology, Wuhan, China; ^2^ Jiangsu Key Laboratory of Marine Pharmaceutical Compound Screening, Jiangsu Key Laboratory of Marine Biological Resources and Environment, Co-Innovation Center of Jiangsu Marine Bio-industry Technology, School of Pharmacy, Jiangsu Ocean University, Lianyungang, China

**Keywords:** SARS-CoV-2, T4 DNA ligase, ligation, recombinase polymerase amplification, nucleic acid detection

## Abstract

The pandemic of COVID-19 caused by severe acute respiratory syndrome coronavirus-2 (SARS-CoV-2) has led to more than 117 million reported cases and 2.6 million deaths. Accurate diagnosis technologies are vital for controlling this pandemic. Reverse transcription (RT)-based nucleic acid detection assays have been developed, but the strict sample processing requirement of RT has posed obstacles on wider applications. This study established a ligation and recombinase polymerase amplification (L/RPA) combined assay for rapid detection of SARS-CoV−2 on genes N and ORF1ab targeting the specific biomarkers recommended by the China CDC. Ligase-based strategies usually have a low-efficiency problem on RNA templates. This study has addressed this problem by using a high concentration of the T4 DNA ligase and exploiting the high sensitivity of RPA. Through selection of the ligation probes and optimization of the RPA primers, the assay achieved a satisfactory sensitivity of 10^1^ viral RNA copies per reaction, which was comparable to RT-quantitative polymerase chain reaction (RT-qPCR) and other nucleic acid detection assays for SARS-CoV−2. The assay could be finished in less than 30 min with a simple procedure, in which the requirement for sophisticated thermocycling equipment had been avoided. In addition, it avoided the RT procedure and could potentially ease the requirement for sample processing. Once validated with clinical samples, the L/RPA assay would increase the practical testing availability of SARS-CoV-2. Moreover, the principle of L/RPA has an application potential to the identification of concerned mutations of the virus.

## Introduction

The pandemic of COVID-19 caused by severe acute respiratory syndrome coronavirus-2 (SARS-CoV-2) has led to more than 117 million reported cases and 2.6 million deaths globally, and the numbers are still increasing (https://www.who.int/). Specific therapeutics, effective vaccines, and accurate diagnosis are vital for control of the pandemic (Jindal and Gopinath, 2020; Liu et al., 2020). Determination of the 29,903-nucleotide (nt), single-stranded RNA (ssRNA) genome sequence of SARS-CoV-2 had made nucleic acid detection of the virus possible (Wu et al., 2020). Based on the sequence information, reverse transcription-quantitative polymerase chain reaction (RT-qPCR) assays were rapidly developed and had become the primary means for detection and diagnosis (Chan et al., 2020; Udugama et al., 2020). The procedure of a typical RT-qPCR assay included converting the viral RNA to complementary DNA (cDNA) and exponential amplification of the detection biomarker with thermal cycling (Feng et al., 2020). Obvious limitations of the RT-qPCR assays were the dependence on sophisticated thermocycling equipment and well-trained personnel. For the diagnosis needs of COVID-19 in resource-limited areas, family self-testing or mass screening, assays based on isothermal amplification technologies like loop-mediated isothermal amplification (LAMP) and recombinase polymerase amplification (RPA) had been developed to reduce the thermocycler dependence (Notomi et al., 2000; Piepenburg et al., 2006; Rabe and Cepko, 2020). Some assays incorporated the clustered regularly interspaced short palindromic repeats (CRISPR) technology to increase the specificity and sensitivity (Broughton et al., 2020; Huang et al., 2020; Zhang et al., 2020). Visualization technologies were applied to read the amplification signals with simple devices or the naked eye, such as portable fluorescence readers or lateral flow strips (LFS) (Behrmann et al., 2020; Zhang C. et al., 2021). These assays provided more choices for practical testing needs of SARS-CoV-2.

**Figure 1:**
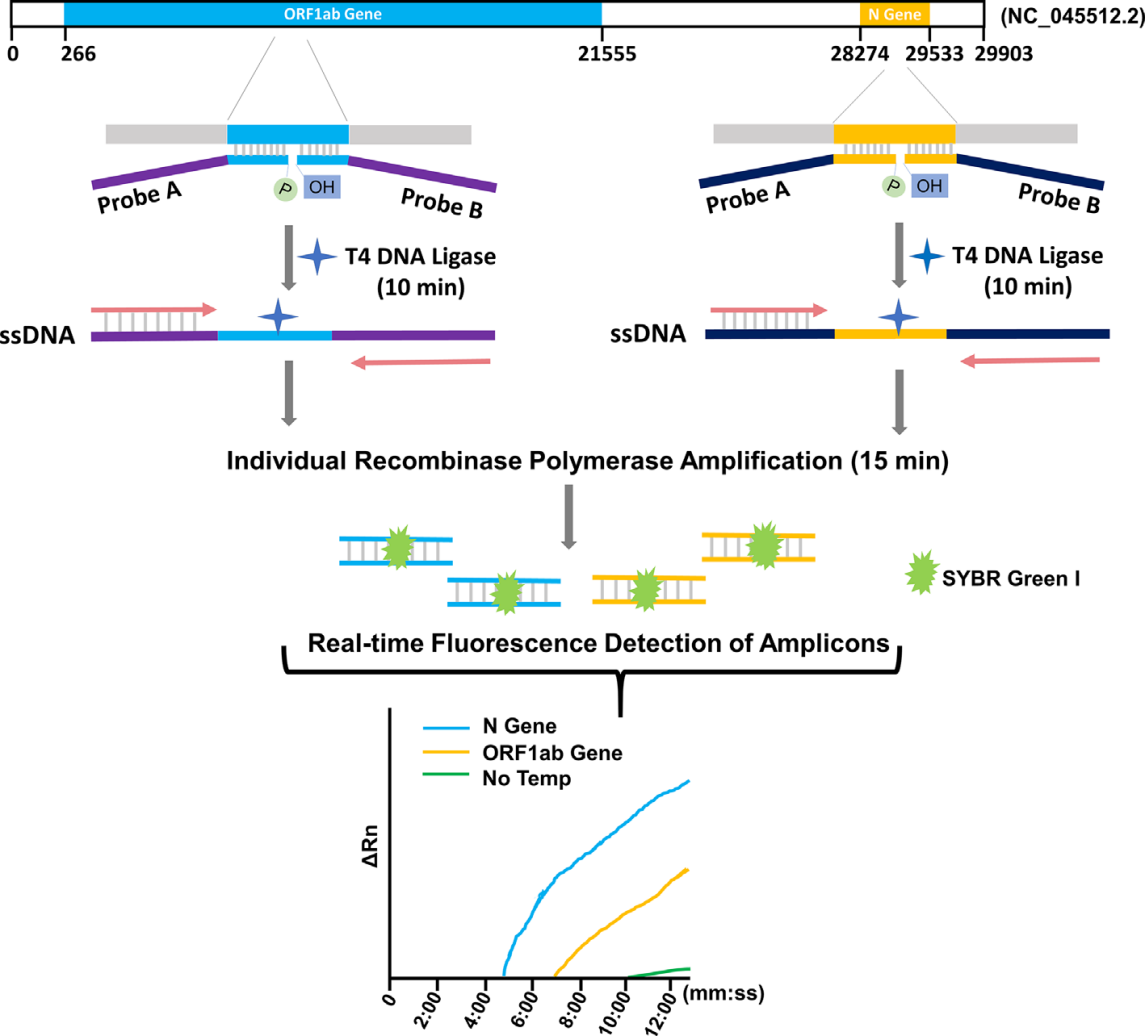
Schematic representation of the L/RPA assay. For each target gene of SRAS-CoV-2 (ORF1ab gene or N gene), an RNA fragment was selected as the detection biomarker. A probe pair (Probe A and Probe B) was designed with the “annealing portions” exactly complementary to the targeted biomarker sequence (indicated with color blue or yellow) and the “amplification arms” completely artificial (indicated with color purple or dark blue). With the presence of the viral RNA, the probe set would anneal to the targeted biomarker and be ligated into one ssDNA fragment with the T4 DNA ligase. The ligation product for each biomarker was individually amplified by RPA with the forward and reverse primers matching the “amplification arm” sequences. With the fluorescence dye SYBR Green I, the amplification signals were detected in real-time. No Temp: the control with no viral RNA template.

Reverse transcription (RT) that converts the viral RNA to cDNA is the fundamental first step of these assays before the nucleic acid amplification, because PCR, LAMP and RPA technologies can only amplify DNA templates. The RT procedure requires high quality of the template RNA as it involves sequential covalent bonding events along the continuous RNA strand. Because RNA is vulnerable to degradation by environmental ribonucleases (RNases), a strict sample processing requirement must be complied with to avoid RNase contamination, which poses obstacles on wide application of the RT-based assays. False negative risk exists when conditions cannot meet the requirement of preparation of RT templates. In this study, we established a ligation and RPA (L/RPA) combined assay for rapid detection of SARS-CoV−2 (**Figure 1**). The L/RPA assay avoided the RT procedure and had a potentially lower operating requirement as compared to RT-based assays. The DNA ligase of bacteriophage T4, an important tool enzyme widely used in molecular cloning and next-generation sequencing (NGS), was the key factor of the assay. The detection biomarkers of the L/RPA assay were RNA fragments on ORF1ab gene and N gene, as recommended by China CDC. For each biomarker, a set of ligation probes (Probe A and Probe B) were designed. Each probe had a portion complementary to the RNA biomarker, and an “amplification arm” to facilitate the RPA amplification. With the presence of viral RNA, the probe set would anneal to the biomarker and be ligated into a single-stranded DNA (ssDNA) fragment by the T4 DNA ligase. The ssDNA fragment would be exponentially amplified in the subsequent isothermal RPA reaction, and the amplification signal could be read in real-time using the SYBR Green I fluorescence dye (**Figure 1**).

Ligase-based strategies had been used for gene rearrangement and single nucleotide polymorphism (SNP) detections (Albrecht et al., 2013; Ruiz et al., 2020), but there had been no report of applications to nucleic acid detection of RNA virus. This was because DNA ligases (e.g., T4 DNA ligase) were not efficient on RNA templates, which could affect sensitivity of the detection (Nilsson et al., 2001; Bullard and Bowater, 2006; Lohman et al., 2014; Wee and Trau, 2016). In this study, we addressed this problem by optimizing the ligation protocol and exploiting the high sensitivity of RPA (Euler et al., 2013; Mayboroda et al., 2018). A satisfactory sensitivity of 10^1^ viral RNA copies per reaction was achieved, which was comparable to that of RT-qPCR. The assay could finish in less than 30 min with a simple procedure. The requirement for sophisticated thermocycling equipment had been avoided. The requirement for sample processing could potentially be eased because the RT procedure had been avoided. Once validated with clinical samples, the L/RPA assay would increase the practical testing availability of SARS-CoV-2. Moreover, the principle of L/RPA has an application potential to the identification of concerned mutations of the virus, which now mainly depends on sequencing.

## Methods

### Design of Probes and Primers

The ligation probes targeted the N gene and the ORF1ab gene of the SARS-CoV-2 genome (GenBank accession no. NC_045512.2). The RNA fragments matching the forward primer, probe, and reverse primer sequences of the RT-qPCR (TaqMan) assay that recommended by the China CDC were selected as the potential detection biomarkers (http://ivdc.chinacdc.cn/kyjz/202001/t20200121_211337.html). Exact complementary sequences of these potential biomarkers were used to design the “annealing portion” of the ligation probes. The “amplification arms” of the probes were artificial sequences that had considered to avoid similarity to nucleic acid sequences of any microorganisms or human by using the NCBI-BLAST. The amplification primers for RPA were determined by the “amplification arm” sequences of the corresponding probes. The general requirements for designing RPA primers were also considered. The Primer Premier 5.0 software was used to analyze the cross-dimerization possibilities between the amplification primers and corresponding ligation probes. The primer and probe sequences are listed in **Supplementary Table 1**. The probes and primers were synthesized by General Biosystems Co Ltd, Anhui, China.

### SARS-CoV-2 Pseudovirus and RNA Extraction

The SARS-CoV-2 pseudovirus (Fubio Biological Technology Co Ltd, Shanghai, China) was composed of a retrovirus capsid with RNA fragments containing the ORF1ab gene, E gene and N gene sequences of SARS-CoV-2. The pseudovirus at a concentration of 10^8^ particles/ml was stored in nuclease-free water under -20°C. RNA in the pseudovirus was extracted with the TIANamp Virus RNA Kit (Tiangen Biotech Co Ltd, Beijing, China) and served as the template.

### RNA Standards of ORF1ab Gene and N Gene Fragments

The pseudovirus RNA was used as the reverse transcription template to synthesize cDNA with the HiScript 1^st^ Strand cDNA Synthesis Kit (Vazyme Biotech Co Ltd, Nanjing, China), and the cDNA was used for PCR amplification to produce DNA fragments of the N gene and the ORF1ab gene. The N gene fragment contained the entire gene sequence, while the ORF1ab gene fragment contained nucleotides from position 13237 to position 13560 of the SARS-CoV-2 genome sequence (GenBank accession no. NC_045512.2), which covered the three potential detection biomarkers on the ORF1ab gene. The PCR products were inserted into pET-28b(+) vector between the T7 promotor and T7 terminator (restriction sites: BamHI/XhoI) to produce the two constructs for transcription of N gene and ORF1ab gene fragments *in vitro*. The two constructs were confirmed by sequencing (General Biosystems Co Ltd). *In vitro* transcription was conducted according to the manufacturer’s instructions of the T7 High Efficiency Transcription Kit (TransGen Biotech Co Ltd, Beijing, China). The transcription products were quantified with a Qubit 4 fluorometer (Thermo Fisher Scientific Inc, Wilmington, DE, USA) and served as the RNA standards. The RNA copy number was calculated based on the transcription size.

### L/RPA Procedure

A 4-µl annealing mixture containing the RNA template and 0.5 µl of each ligation probe (1 µM) was heated to 85°C for 2 min and cooled on ice to anneal the probes to the template. Then 0.5 µl of the 10X T4 DNA ligase buffer and 0.5 µl (equal to 40-500 cohesive end units according to the enzyme concentration) of the T4 DNA ligase (Thermo Fisher Scientific Inc) were added to the mixture. Ligation was done in 8-10 min at 37°C and inactivated at 95°C for 2 min. The whole ligation mixture was added to the RPA mixture of the RAA-Basic Nucleic Acid Amplification Reagent (Hangzhou ZC Bio-Sci & Tech Co Ltd, Hangzhou, China) containing 2 µl of each primer (10 µM), 36 µl of A Buffer and 2.5 µl of B Buffer (Hangzhou ZC Bio-Sci & Tech Co Ltd). SYBR Green I fluorescent dye (10,000X) (Beijing Solarbio Science & Technology Co Ltd, Beijing, China) was diluted to 15X by A Buffer and 2.5 µl was used in each RPA reaction (total volume 50 µl). The reaction was conducted on an Applied Biosystems 7900HT Fast Real-Time PCR System at 37°C with signal reads at 20-sec intervals for 15 min.

### RT-qPCR

The RT-qPCR reactions were performed according to the manufacturer’s instructions of the Novel Coronavirus (2019-nCoV) Dual Probes qRT-PCR Kit (Beyotime Biotechnology Inc, Shanghai, China). The primer and probe sequences were designed according to the China CDC’s recommendation (**Supplementary Table 1**). A total of 5 µl of the RNA template was added to the reaction premix to make the total reaction volume to 25 µl. Reactions were conducted on an Applied Biosystems 7900HT Fast Real-Time PCR System. The cycle setting was 20 min at 50°C for reverse transcription, 2 min at 95°C for denaturation, followed by 45 cycles of 15 sec at 95°C and 20 sec at 60°C. The fluorescence channels were VIC for ORF1ab gene and FAM for N gene.

## Results

### Selection of the Detection Biomarkers

For this study, potential detection markers were the RNA fragments of the virus genome matching the forward primer, probe, and reverse primer sequences of RT-qPCR (TaqMan) that recommended by the China CDC for detection of SARS-CoV−2 (**Supplementary Table 1**). Thus, there were three potential detection biomarkers on the N gene, and another three on the ORF1ab gene. For each potential biomarker, a set of two ligation probes (Probe A and Probe B) was designed (**Figure 1**). More specifically, for N gene, N-Probe 1A and 1B targeted the fragment matching the qPCR forward primer sequence; N-Probe 2A and 2B targeted the fragment matching the qPCR probe sequence; and N-Probe 3A and 3B targeted the fragment matching the qPCR reverse primer sequence. Similarly, a series of O-Probes were designed for the potential biomarkers on the ORF1ab gene (**Supplementary Table 1**).

The ligation efficiency of the L/RPA procedure was fundamental for the overall performance of the detection. A series of preliminary experiments were conducted to increase the ligation efficiency and found that the concentration of T4 ligase in the ligation system was a key factor. It was determined to use 500 U (cohesive end units) of T4 ligase in the 5-μl ligation mixture (**Supplementary Table 2**). Briefly, 40 U, 200 U and 500 U of T4 ligase per reaction were tested for both N gene and ORF1ab gene. For N gene, we used N-Probe 2A and 2B for ligation and the amplification primer set N-Primer 1F and 1R for RPA. For ORF1ab gene, we used O-Probe 1A and 1B and O-Primer 1F and 1/2R. According to the principle, the ligation probes shared the same sequences of the “amplification arms” that matched the amplification primer set (**Supplementary Table 1**). Different template amounts (10^7^, 10^3^ and 10^1^ copies) were tested and the Threshold time (*Tt*) of the fluorescent signals were compared with the reactions with no template (No Temp). The differences of *Tt* (Δ*Tt*) between the reaction with no template and the reactions with various amounts of template reflected the performance of the assay. For both genes, acceptable Δ*Tt* were obtained for the various template amounts when 500 U/reaction of T4 ligase was used. Poor Δ*Tt* were observed for 10^3^ or 10^1^ copies of template when 200 U or 40 U of T4 ligase per reaction was used (**Supplementary Table 2**). Thus, it was determined to use 500 U/reaction of T4 ligase in this assay.

**Figure 2:**
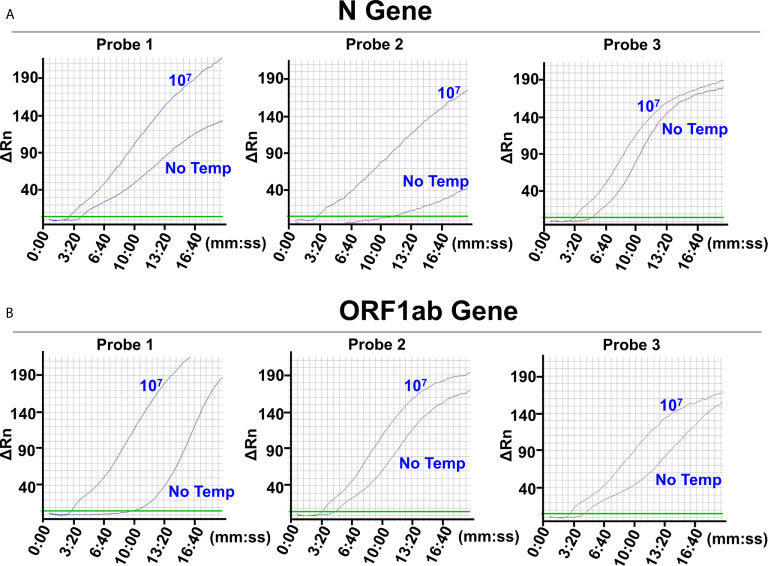
Selection of the detection biomarkers. The images were the fluorescence history diagrams of RPA amplifications of the ligation products from the N gene RNA standard template **(A)** and the ORF1ab gene RNA standard template **(B)**. The diagrams showed the fluorescence signal (ΔRn) *vs*. time (mm:ss). For each target gene, 3 potential detection biomarkers were targeted by ligation probe pairs 1, 2, and 3 that were indicated at the top of the respective diagrams. The curves indicated with “10^7^” represented amplification signals from ligations with 10^7^ the RNA templates. The curves indicated with “No Temp” represented amplification signals from the ligation controls with no RNA template. Every diagram was one typical outcome of three independent experiments.

To select the biomarker for the N gene, probe pairs 1 (N-Probe 1A and 1B), 2 (N-Probe 2A and 2B), and 3 (N-Probe 3A and 3B) were tried in the L/RPA reactions using the same amplification primer set (N-Primer 1F and 1R) (**Supplementary Table 1**). The amplification signals of reactions with 10^7^ N gene copies and reactions with no template were compared for each probe pair. The probe pair 2 produced the most distinct signals (the most different *Tt* values) between reactions with 10^7^ N gene copies and reactions with no template, suggesting that the RNA fragment on the N gene matching the qPCR probe sequence should be selected as the biomarker for the N gene (**Figure 2A** and **Supplementary Table 3**).

Similarly, to select the biomarker for the ORF1ab gene, O-Probe 1A and 1B, 2A and 2B, and 3A and 3B were tried in the L/RPA reactions using O-Primer 1F and 1/2R (**Supplementary Table 1**). The probe pair 1 produced the most distinct signals and the most different *Tt* values between reactions with 10^7^ ORF1ab gene copies and reactions with no template, suggesting that the RNA fragment on the ORF1ab gene matching the qPCR forward primer sequence should be selected as the biomarker for the ORF1ab gene (**Figure 2B** and **Supplementary Table 3**).

### Screening for Optimal RPA Primer Sets

**Figure 3:**
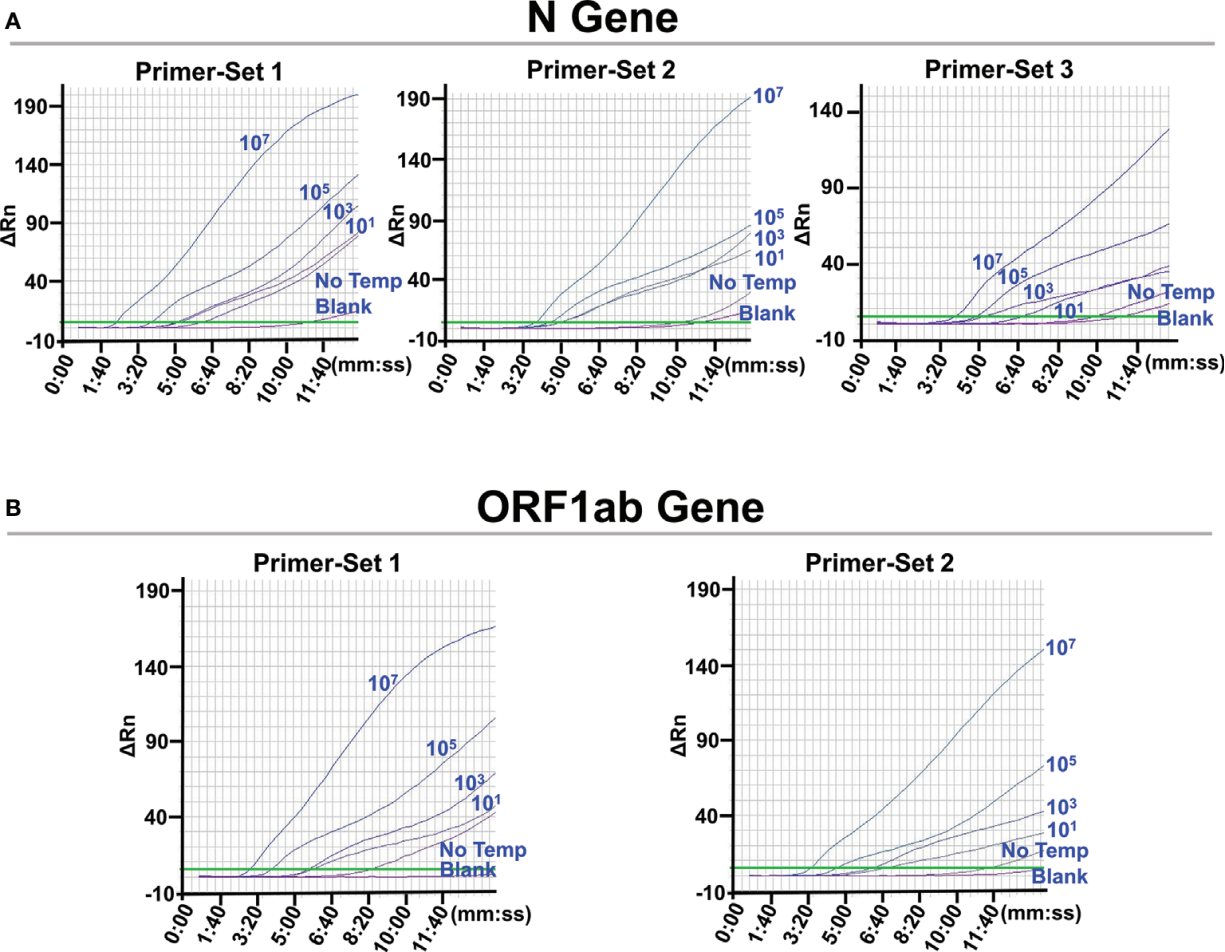
Screening for optimal RPA primer sets. The images were the fluorescence history diagrams of RPA amplifications with different primer sets for the detection of N gene **(A)** and ORF1ab gene **(B)**. For RPA reactions using each primer set, the corresponding probe pairs were used for the preceding ligation reactions. The diagrams showed the fluorescence signal (ΔRn) *vs*. time (mm:ss). The blue numbers beside the curves indicated the amount of RNA standard templates (in copies) used in the corresponding L/RPA assays. No Temp: the control with no RNA template. Blank: the control RPA reactions without the ligation reaction components and the RNA template. Every diagram was one typical outcome of three independent experiments.

The limit of detection (LOD) of the L/RPA assay for the N gene using the probe pair 2 (N-Probe 2A and 2B) and primer set 1 (N-Primer 1F and 1R) was tested with series dilutions of the N gene RNA fragments. The results showed close signal curves and *Tt* values of reactions with 10^3^ copies, 10^1^ copies, and no template (**Figure 3A** and **Supplementary Table 4**). Similar results were obtained from the L/RPA assay for the ORF1ab gene detection using the probe pair 1 (O-Probe 1A and 2B) and primer set 1 (O-Primer 1F and 1/2R) (**Figure 3B** and **Supplementary Table 4**).

For better LOD, signals of reactions with no template should appear later and distinct from reactions with 10^1^ copies of template. Possible cross-dimerization between the amplification primers and ligation probes were analyzed with the Primer Premier 5.0 software and additional probe and primer sets were designed (**Supplementary Table 1**). For the N gene, one additional forward primer (N-Primer 2/3F) and two additional reverse primer (N-Primer 2R and 3R) were designed and used as Primer-Set 2 (N-Primer 2/3F and 2R) and Primer-Set 3 (N-Primer 2/3F and 3R). Because the “amplification arm” sequences of the ligation probes should match the amplification primer sequences, N-Probe 2A-2/3, N-Probe 2B-2, and N-Probe 2B-3 were designed and used together with N-Primer 2/3F, N-Primer 2R, and N-Primer 3R, respectively. For the ORF1ab gene, one additional forward primer (O-Primer 2F) was designed and used in Primer-Set 2 (O-Primer 2F and 1/2R). O-Probe 1A-2 was designed and used together with O-Primer 2F.

These newly designed primer sets showed later signals and bigger *Tt* values of the no template reactions that led to better LOD. For the N gene, Primer-Set 2 showed close signal curves of reactions with 10^3^ and 10^1^ copies of template, and Primer-Set 3 was selected (**Figure 3A** and **Supplementary Table 4**). For the ORF1ab gene, Primer-Set 2 was selected (**Figure 3B** and **Supplementary Table 4**). For both the N gene and the ORF1ab gene, the L/RPA assay exhibited a LOD of 10^1^ copies per reaction.

The finally selected probes and primers in the L/RPA assay were listed in **Supplementary Table 1** with the primer/probe names indicated in red. The sequences were as follows (5’-3’, the complementary portions of the probes were italicized):

N-Probe 2A-2/3, ***GCAGCAGCAA***GACGAGGGAAAGAGCAGTACCTAA;
N-Probe 2B-3, TGTGTACGAATCCCACTAATTCGCC***AATCTGTCAA***;
N-Primer 2/3F, TTAGGTACTGCTCTTTCCCTCGTC;
N-Primer 3R, TGTGTACGAATCCCACTAATTCGCC;
O-Probe 1A-2, ***AACCCACAGGG***CAATAGGGAGATCATAGGAGTTGGCT;
O-Probe 1B, TAACTCATATTGTAGAAGAGTAGAAG***TTAAGTGTAA***;
O-Primer 2F, AGCCAACTCCTATGATCTCCCTATTG;
O-Primer 1/2R, TAACTCATATTGTAGAAGAGTAGAAG.

### Validation With SARS-CoV-2 Pseudovirus

**Figure 4:**
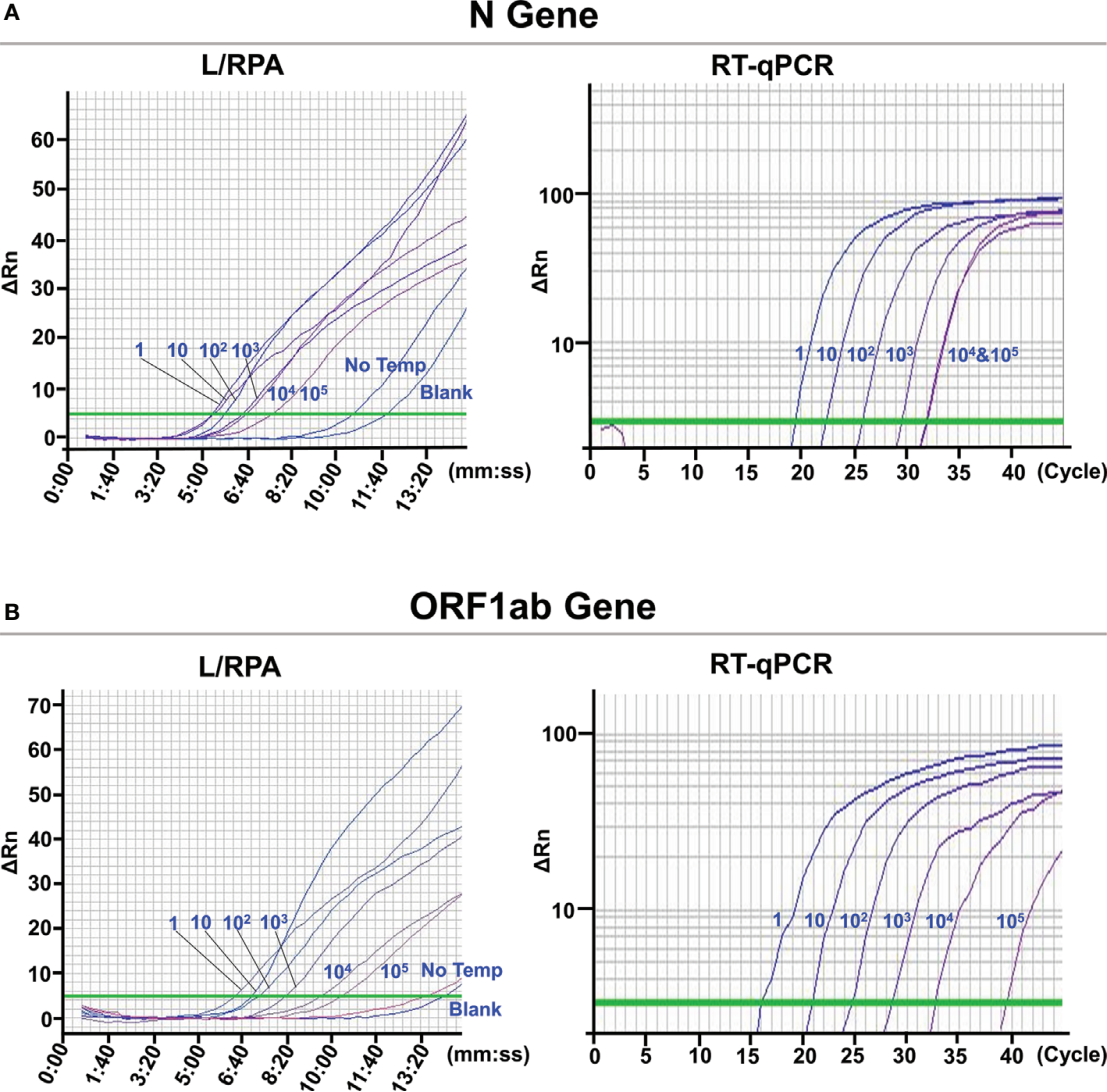
Validation with SARS-CoV-2 pseudovirus. The images were the fluorescence history diagrams of the detection results of the N gene **(A)** and the ORF1ab gene **(B)** of SARS-CoV-2 pseudovirus using the L/RPA assay and RT-qPCR. The diagrams of L/RPA showed the fluorescence signal (ΔRn) *vs*. time (mm:ss). The diagrams of RT-qPCR showed the fluorescence signal (ΔRn) *vs*. *Ct* (cycle). The blue numbers beside the curves indicated the dilution multiple of the RNA extracted from the pseudovirus used in the corresponding assays. No Temp: the control with no RNA template. Blank: the control reactions without the ligation reaction components and the RNA template. Every diagram was one typical outcome of three independent experiments.

The L/RPA assay was validated with SARS-CoV-2 pseudovirus. RNA extracted from the SARS-CoV-2 pseudovirus was 10-fold serially diluted and detected by the L/RPA assay for both the N gene and the ORF1ab gene. The results were compared with the RT-qPCR method (**Figure 4** and **Supplementary Table 5**). For both genes, up to dilution multiple of 10^5^, the L/RPA assay could give positive signals. At this dilution multiple, the RT-qPCR detection gave a non-linear signal for the N gene, and gave a signal in the range of “suspected” for the ORF1ab gene, which suggested that the template amount was close to the detection limit. Thus, the sensitivity of L/RPA assay was at the same level as RT-qPCR.

## Discussion

Rapid and simple SARS-CoV-2 detection methods that are not limited by sophisticated thermocycling equipment are valuable for diagnosis needs in resource-limited areas, family self-testing or mass screening. For this purpose, assays based on LAMP, RPA and CRISPR technologies have been developed (Rabe and Cepko, 2020; Haq et al., 2021; Zhang W. S. et al., 2021). These assays are generally faster and simpler than RT-qPCR, providing more choices for the testing needs of SARS-CoV-2 under different situations. Reverse transcription (RT) is the fundamental first step of these assays that converts the viral RNA to cDNA, the initial material for nucleic acid amplification. Because of the ubiquitous environmental RNase, the RT procedure has strict operating requirements for RNase-free environment, containers, and reagents, which complicate the application of RT-based assays. The L/RPA assay established in this study avoided the RT procedure. Instead, two ligation probes were annealed to adjacency on the RNA template and ligated into a ssDNA fragment for subsequent amplification. This strategy used the RNA template only for the ligation, a “one covalent bonding” event that was quicker and much simpler than RT. The fewer RNA involvement should potentially ease the operating requirement of the whole assay.

This is the first application of ligase-based strategies to the detection of RNA virus. Because the T4 DNA ligase is inefficient on RNA templates (Nilsson et al., 2001; Bullard and Bowater, 2006), it is important to bring up the ligation efficiency to the level that exponential amplification of the ligation product can occur. During the optimization of the ligation protocol, we found that using small reaction volume and keeping high concentration of the T4 DNA ligase were the key factors. It was determined that using 500 U of the T4 ligase in a 5-μl reaction mixture could achieve sufficient ligation efficiency. On the other hand, the highly sensitive RPA technology had to be used, while PCR was not sensitive enough to guarantee a satisfactory sensitivity of the assay.

The detection biomarkers of the L/RPA assay were selected from the viral RNA fragments matching the forward primer, probe, and reverse primer sequences of RT-qPCR (TaqMan) that recommended by the China CDC. One biomarker was selected for each of the target genes, N and ORF1ab. This ensured the good specificity of the selected biomarkers towards SARS-CoV-2, because the sequences had been used in hundreds of millions of clinical detections worldwide in RT-qPCR assays. For each biomarker, sequence optimizations were carefully conducted for the L/RPA assay. The sequence optimization process of this study suggested that the sequences of the “amplification arms” of the probes were important. Inappropriate sequences could possibly form cross-dimers between the amplification primers and ligation probes and produce noise fluorescence signals, affecting the sensitivity.

The L/RPA assay showed a sensitivity of 10^1^ viral RNA copies per reaction, which was comparable to that of RT-qPCR and other nucleic acid detection assays for SARS-CoV-2 (Behrmann et al., 2020; Zhang C. et al., 2021; Zhang W. S. et al., 2021). The assay could be finished in less than 30 min with simple operation and without dependence on sophisticated thermocycling equipment. Because of the enzyme characteristics, both the ligation and the RPA reactions does not demand strict temperature control, and the set temperature of 37°C can even be provided by the body heat. Although a qPCR machine was used in this study to read the fluorescence signal, the thermocycling function had been avoided. Fluorescent signal detection of SYBR Green I DNA staining is a mature technology, and battery-powered portable tube scanners are commercially available and ready to be applied to the fluorescence detection of the L/RPA assay (Mondal et al., 2016).

Though the sensitivity of 10^1^ viral RNA copies per reaction was comparable to RT-qPCR, our results did not show a good relationship between the *Tt* value and the template amount. This means quantitative detection of SARS-CoV-2 could not be achieved by the L/RPA assay. One possible reason was that the amplification substrate was the ligation product of the two ligation probes, and the ligation efficiency was not linearly related to the template amount. Considering the major need of practical SARS-CoV-2 tests was to give an answer of positive or negative, the L/RPA assay without the capacity of quantification was still a competent detection choice. Based on the results of this study, a tentative evaluation standard for both N gene and ORF1ab gene could be: Δ*Tt* ≥ 2.0 min, positive; 2.0 min > Δ*Tt* ≥ 0.5 min, suspected; Δ*Tt* < 0.5 min, negative; where Δ*Tt* = *Tt*_No Temp_ – *Tt*_Sample_. As a future direction, validation of the L/RPA assay with clinical samples should be a requirement before it can be applied to practical testing, and a more accurate evaluation standard should be determined based on the detection of adequate clinical samples.

In conclusion, this study established a ligation-based L/RPA assay for rapid detection of SARS-CoV−2. The assay is more easily operated than the RT-based assays because the RNA involvement has been reduced. Not limited by sophisticated thermocycling equipment, the assay provides more choice for the detection of SARS-CoV−2 at point of need. Furthermore, the principle of the assay is readily applicable to the identification of concerned mutations of the virus, which now mainly depends on sequencing.

## Data Availability Statement

The original contributions presented in the study are included in the article/**Supplementary Material**, further inquiries can be directed to the corresponding authors.

## Author Contributions

QL, YC, and SG designed the research. PW, CM, XZ, LC, and LY conducted the research. PW, CM, XZ, and XL analyzed the data. PW and SG wrote the manuscript. All authors contributed to the article and approved the submitted version.

## Funding

This work was supported by grants from the National Natural Science Foundation of China (31470275), the Key Natural Science Research Project of the Jiangsu Higher Education Institutions of China (20KJA416002), the Lianyungang Science and Technology Project of China (SF2003), the Science and Technology Project of Lianyungang High-tech Zone of China (HZ201901), the Open-end Funds of Jiangsu Key Laboratory of Marine Pharmaceutical Compound Screening (HY202004), and the Priority Academic Program Development of Jiangsu Higher Education Institutions of China.

## Conflict of Interest

The authors declare that the research was conducted in the absence of any commercial or financial relationships that could be construed as a potential conflict of interest.
