# Supplementary material for: A Ligation/Recombinase Polymerase Amplification Assay for Rapid Detection of SARS-CoV−2
Source: Front Cell Infect Microbiol. 2021 May 28;11:680728. doi: 10.3389/fcimb.2021.680728 (PMC8193850; doi:10.3389/fcimb.2021.680728)
Supplement: Supplementary file 1 [file DataSheet_1.pdf]

Supplementary Material

A ligation/recombinase polymerase amplification assay for rapid detection of SARS-CoV-2

Pei Wang[1,†], Chao Ma[2,†], Xue Zhang[2,†], Lizhan Chen[2], Longyu Yi[1], Xin Liu[1], Qunwei Lu[1,*], Yang Cao[2,*] and Song Gao[2,*]

[1]Key Laboratory of Molecular Biophysics of Ministry of Education, Department of Biomedical Engineering, College of Life Science and Technology, Center for Human Genome Research, Huazhong University of Science and Technology, Wuhan 430074, China

[2]Jiangsu Key Laboratory of Marine Pharmaceutical Compound Screening, Jiangsu Key Laboratory of Marine Biological Resources and Environment, Co-Innovation Center of Jiangsu Marine Bio-industry Technology, School of Pharmacy, Jiangsu Ocean University, Lianyungang 222005, China

[†]These authors contributed equally to this work.

*Correspondence:

Qunwei Lu luqw@hust.edu.cn

Yang Cao 2020000088@jou.edu.cn

Song Gao gaos@jou.edu.cn

Supplementary Table 1. Primers and Probes used in this study.

Assay	Target gene	Primer/probe name	Sequence (5'-3')	Length (nt)
L/RPA	N	N-Probe 1A	**PHO-**AGAAGTTCCCCGACGAGGGAAAGAGTTGTACCTAAAT	37
		N-Probe 1B	TGTATAGGAATCCCACTGAATTTTTCATTCTAGCAGG	37
		N-Probe 2A	**PHO-**GCAGCAGCAAGACGAGGGAAAGAGTTGTACCTAAAT	36
		**N-Probe 2A-2/3**	**PHO-**GCAGCAGCAAGACGAGGGAAAGAGCAGTACCTAA	34
		N-Probe 2B	TGTATAGGAATCCCACTGAATTTTTCAATCTGTCAA	36
		N-Probe 2B-2	TGTGTACGAATCCCACAAGAATCAATCTGTCAA	33
		**N-Probe 2B-3**	TGTGTACGAATCCCACTAATTCGCCAATCTGTCAA	35
		N-Probe 3A	**PHO-**TCTCAAGCTGGACGAGGGAAAGAGTTGTACCTAAAT	36
		N-Probe 3B	TGTATAGGAATCCCACTGAATTTTTCCAGACATTTTGC	38
		N-Primer 1F	ATTTAGGTACAACTCTTTCCCTCGTC	26
		N-Primer 1R	TGTATAGGAATCCCACTGAATTTTTC	26
		**N-Primer 2/3F**	TTAGGTACTGCTCTTTCCCTCGTC	24
		N-Primer 2R	TGTGTACGAATCCCACAAGAATC	23
		**N-Primer 3R**	TGTGTACGAATCCCACTAATTCGCC	25
	ORF1ab	O-Probe 1A	**PHO-**AACCCACAGGGGATAAGGGAGATAATAAGAGTTGGGT	37
		**O-Probe 1A-2**	**PHO-**AACCCACAGGGCAATAGGGAGATCATAGGAGTTGGCT	37
		**O-Probe 1B**	TAACTCATATTGTAGAAGAGTAGAAGTTAAGTGTAA	36
		O-Probe 2A	**PHO-**ACATACCGCAGACGGGATAAGGGAGATAATAAGAGTTGGGT	41
		O-Probe 2B	TAACTCATATTGTAGAAGAGTAGAAGCCATAACCTTTCC	39
		O-Probe 3A	**PHO-**CATCAGCTGAGATAAGGGAGATAATAAGAGTTGGGT	36
		O-Probe 3B	TAACTCATATTGTAGAAGAGTAGAAGACGATTGTG	35
		O-Primer 1F	ACCCAACTCTTATTATCTCCCTTATC	26
		**O-Primer 1/2R**	TAACTCATATTGTAGAAGAGTAGAAG	26
		**O-Primer 2F**	AGCCAACTCCTATGATCTCCCTATTG	26
RT-qPCR (TaqMan)	N	Forward Primer	GGGGAACTTCTCCTGCTAGAAT	22
		Probe	**FAM**-TTGCTGCTGCTTGACAGATT-**TAMRA**	20
		Reverse Primer	CAGACATTTTGCTCTCAAGCTG	22
	ORF1ab	Forward Primer	CCCTGTGGGTTTTACACTTAA	21
		Probe	**VIC**-CCGTCTGCGGTATGTGGAAAGGTTATGG-**BHQ1**	28
		Reverse Primer	ACGATTGTGCATCAGCTGA	19

Supplementary Table 2. Screening for the T4 ligase concentration.

**Biomarker**	**Template** (Copies)	$\Delta Tt = Tt_{No\ Temp} - Tt_{Template}$ (min)		
		**40 U/reaction**	**200 U/reaction**	**500 U/reaction**
**N Gene** (Probe 2)	10^7	3.85±0.27	3.96±0.17	3.56±0.42
	10^3	0.02±0.42	0.15±0.54	4.37±0.39
	10^1	-0.53±0.31	-0.38±0.30	1.75±0.26
**ORF1ab Gene** (Probe 1)	10^7	4.00±0.52	4.61±0.43	3.99±0.31
	10^3	0.62±0.44	0.94±0.19	1.56±0.37
	10^1	0.30±0.52	-0.22±0.36	1.48±0.28

Supplementary Table 3. Threshold time (*Tt*) of the probe screening.

**Template** (Copies)	**N Gene Probes** (mm:ss)			**ORF1ab Gene Probes** (mm:ss)		
	1	**2**	3	**1**	2	3
10^7	2:50±0:43	3:06±0:29	3:14±0:31	3:04±0:19	3:24±0:54	2:48±0:25
No Temp	4:12±0:52	11:16±0:36	5:10±0:26	9:48±0:27	4:50±0:49	4:26±0:52

Supplementary Table 4. Threshold time (*Tt*) of the primer set screening.

**Template** (Copies)	**N Gene Primer Sets** (mm:ss)			**ORF1ab Gene Primer Sets** (mm:ss)	
	1	2	**3**	1	**2**
10^7	2:20±0:40	3:54±0:31	4:00±0:25	2:32±0:33	3:12±0:29
10^5	3:58±0:33	4:32±0:25	4:54±0:29	3:42±0:26	4:22±0:27
10^3	5:10±0:24	5:02±0:38	5:16±0:27	5:18±0:36	5:50±0:30
10^1	5:18±0:23	5:04±0:27	6:52±0:39	5:28±0:39	6:26±0:20
No Temp	6:16±0:43	10:12±0:26	9:42±0:28	7:56±0:41	10:46±0:25
Blank	11:03±0:39	11:08±0:40	11:04±0:42	15:28±0:42	15:24±0:35

Supplementary Table 5. Detection of SARS-CoV-2 pseudovirus.

**Dilution Multiple**	**N Gene**		**ORF1ab Gene**	
	L/RPA (*Tt*)	RT-qPCR (*Ct*)	L/RPA (*Tt*)	RT-qPCR (*Ct*)
1	5:22±0:23	19.41±0.22	6:24±0:30	16.05±0.13
10	5:24±0:18	22.44±0.16	7:02±0:26	21.17±0.15
10^2	5:52±0:25	25.92±0.28	7:18±0:28	24.96±0.23
10^3	6:28±0:30	29.63±0.21	8:20±0:23	28.37±0.25
10^4	6:40±0:28	32.03±0.19	9:40±0:23	33.72±0.22
10^5	7:36±0:33	32.04±0.27	10:24±0:19	39.38±0.26
No Temp	10:38±0:27	Not Applicable	13:36±0:34	Not Applicable
Blank	11:52±0:43	Not Detected	14:14±0:28	Not Detected
